# Mortality and dementia in untreated iNPH: a 25-year follow-up of a population-based cohort

**DOI:** 10.1186/2045-8118-12-S1-O7

**Published:** 2015-09-18

**Authors:** Daniel Jaraj, Katrin Rabiei, Thomas Marlow, Christer Jensen, Ingmar Skoog, Carsten Wikkelsø

**Affiliations:** 1 Inst of Neuroscience and Physiology, University of Gothenburg, Sweden; 2 Inst of Clinical Sciences, University of Gothenburg, Sweden

## Introduction

We examined mortality and risk of dementia in persons with untreated possible and probable iNPH using a large, prospective population-based cohort.

## Methods

1235 persons aged 70 years or more were included. Baseline examinations, including CT of the brain, were made between 1986 and 2000. Cases were diagnosed using criteria from international consensus guidelines and were followed until 2012. A total of 53 persons had radiological features compatible with iNPH. Of these, 24 fulfilled criteria for probable iNPH, while 29 were asymptomatic or had possible iNPH. None of the cases had been treated with shunt. Outcome data was obtained from clinical examinations, the Swedish Hospital Discharge Register and the National Swedish Death Registry. Risks were compared using Cox proportional hazard regression.

## Results

Median follow-up time was 11.5 years (Maximum 25 years). Crude 5-year mortality was 88.5 % in those with probable iNPH (n=24), and 19.1 % in those without iNPH (p<0.001). Adjusting for age, sex and cohort, mortality was increased throughout follow-up among persons with probable iNPH (Hazard Ratio 3.8; 95% CI: 2.5-6.0). Main causes of death were cardiovascular disorders. Among those with possible iNPH and asymptomatic radiological iNPH (n=29), who did not have dementia at baseline, 40 % developed dementia during follow-up (Hazard Ratio 2.6; 95% CI: 1.3-5.1).

## Conclusions

The risk of mortality is substantially increased in those with untreated iNPH. Our findings indicate that persons with radiological features of iNPH have an increased risk of dementia even if they do not fulfill the current clinical criteria for probable iNPH.
